# Fluid Shear Stress Enhances the Phagocytic Response of Astrocytes

**DOI:** 10.3389/fbioe.2020.596577

**Published:** 2020-11-11

**Authors:** Nicole M. Wakida, Gladys Mae Saquilabon Cruz, Pegah Pouladian, Michael W. Berns, Daryl Preece

**Affiliations:** ^1^Berns Laboratory, Beckman Laser Institute and Medical Clinic, University of California, Irvine, Irvine, CA, United States; ^2^Preece Laboratory, Beckman Laser Institute and Medical Clinic, University of California, Irvine, Irvine, CA, United States

**Keywords:** astrocyte, phagocytosis, shear stress, mechanotransduction, laser nanosurgery, laser ablation

## Abstract

Astrocytes respond to brain injury at a cellular level by the process of reactive astrogliosis, and are able to adjust their response according to the severity of the insult. Included in the reactive response is the process of phagocytosis, where astrocytes clean up surrounding cellular debris from damaged cells. In this study, we observe the process of phagocytosis by primary cortical astrocytes in the presence of media flow across the apical surface of the cells. Both static and cells under flow conditions respond consistently via phagocytosis of laser-induced cellular debris. We found that astrocytes exposed to shear flow initiate phagocytosis at a consistently faster rate than cells observed under static conditions. Shear forces created by laminar flow were analyzed as well as the flow fields created around astrocyte cells. Results suggest astrocyte phagocytosis is a mechanosensitive response, thus revealing the potential to enhance astrocyte phagocytic cleanup of damaged nervous tissue.

## Introduction

Astrocytes have the ability to respond to nervous tissue damage by the process of reactive astrogliosis. This topic has been of growing importance, as it holds the potential to mitigate the spread of brain injury. The functional response of reacting astrocytes vary depending on the severity of the insult to the CNS. Included in the response to nervous tissue damage is morphological changes of astrocytes and the uptake of extruded material (Sofroniew, 2009).

The study of mechanotransduction, converting mechanical forces into cellular signals, has revealed that membrane receptors can transduce external forces to cellular changes via signaling. These mechanosensors are believed to stimulate a variety of cellular responses ranging from cell proliferation, to contraction, and remodeling of the cell cytoskeleton (Sun et al., 2016). Studies on cellular response to shear stress have extensively focused on endothelial cells, as blood flowing through vessels continually exerts force on endothelial cells lining vasculature (White and Frangos, 2007). The ability to sense and respond to changes in blood flow is a necessity to maintain homeostasis, and has been implicated in a role in the progression of Atherosclerosis (Gimbrone et al., 1997). Mechanical forces resulting from shear stress have been shown to modulate developmental processes in addition to homeostasis in endothelial cells (Potter et al., 2014). Although most shear stress studies focus on endothelial cells, macrophages have also been studied. Mazur et al. stimulated macrophages by fluid shear stress and reported an increased deformation of cell shape (Mazur and Williamson, 1977). These macrophages were additionally reported to retain the ability to phagocytose polystyrene beads. With the increase in interest in astrocytes, a few studies focusing on astrocytes subjected to shear flow conditions have been recently published that generates high-speed shear forces to model TBI-like low-amplitude shear forces (Maneshi et al., 2015, 2017, 2018). Maneshi et al. (2018) demonstrate redistribution of cytoskeletal elements in response to fluid shear stress exposure to astrocytes. This observation in astrocytes is mimicked in endothelial cells responding to slow shear flow with increased traction forces and cell-cell stresses (Perrault et al., 2015).

For many years, astrocytes were believed to function passively within the Central Nervous System (CNS) (Markiewicz and Lukomska, 2006). Recent studies have demonstrated the dynamic nature of astrocytes, including the ability to phagocytose cellular debris (Lööv et al., 2012; Wakida et al., 2018) and to modulate synapse structure and function (Ridet et al., 1997). Additional studies have demonstrated astrocytes and microglia working collectively to limit tissue degeneration after disease and injury as protection for neuron function (Sofroniew, 2005; Khakh and Sofroniew, 2015; Liddelow et al., 2017). A growing number of studies implicate astrocytes’ importance in maintaining tissue homeostasis, demonstrating the need for a better understanding of the effect extracellular factors have on their cellular activity. This is of critical importance to areas of research like traumatic brain injury and CNS repair. Our previous studies demonstrated the ability of astrocytes to respond to laser induced cell lysis via phagocytosis (Wakida et al., 2018, 2020). Membrane ruffling and subsequent vesicle formation in the responding astrocyte was associated with cellular debris from the targeted cell with the use of propidium iodide labeled DNA and pHrodo dye. Here, our goal is to determine if the phagocytic response in astrocytes is activated or enhanced by mechanical or chemical component of photolysis by laser ablation/nanosurgery.

## Methods

### Primary Astrocyte Cultures

Astrocytes were dissociated from E18 mouse cortical tissue acquired from Brain Bits, LLC. (Brain Bits animal protocol #32-08-013 was approved by the Southern Illinois University School of Medicine Laboratory Animal Care and Use Committee on 5/18/11). Cells were dissociated by an 8 min incubation with 2 mg/mL papain (Sigma) in Hibernate E (without Calcium and B27, BrainBits) at 37°C. Astrocytes were resuspended into Co-culture media (BrainBits) and plated onto matrigel (Corning) coated u-Slide I 0.6 Luer (Ibidi, Inc.) at a concentration of 1 × 10^6^ cells/mL. The channel of the U-slide was coated with polylysine and Matrigel (Wakida et al., 2018). Cells were incubated at 37°C with 5% CO_2_ under static conditions.

### Shear flow

Astrocytes (GFAP positive via antibody staining) were plated within the u-Slide for a minimum of 48 h prior to observation. Cells were incubated with an Ibidi stage incubation unit with controlled temperature at 37°C, humidity at 70%, and 5% CO_2_ levels. An Ibidi pump system, including the pump and fluidic units, allowed for coculture media to flow through the channel of the u-Slide in combination with an Ibidi heating unit. Ibidi pump system flowed media over cells immediately following laser exposure at a flow rate of flow rate 0.8 mL/min (2 mbar pressure 0.48 dyn/cm^2^).

### Laser Microirradiation

An inverted Zeiss Axiovert 200M microscope in conjunction with a Hamamatsu CCD camera was used to acquire phase contrast images at 2 s intervals. Images were acquired using a 63x 1.4 NA oil immersion objective. The nucleus of astrocytes was targeted with an 800 nm Coherent Mira femtosecond laser, as described in Wakida et al. (2018). The response of adjacent cells sharing a membranous connection with irradiated cells was observed. Initiation of phagocytosis was determined by increased membrane ruffling and endocytic vesicle formation at the cell periphery, near the laser induced cell debris. The combined setup of the pump system, laser optical path, and microscope are diagramed in Supplementary Figure 1.

### Quantitative Phase Microscopy

To estimate the forces on an average astrocytes, the average height of the observed astrocytes was required. To do this, images of 16 astrocytes were taken using a Hamamatsu Quantitative Phase Microscope (QPM) with a 40x objective (Yamauchi et al., 2011). The QPM retrieves the phase retardance of light passing through cell. Assuming a constant refractive index, it was possible to calculate the thickness of the cell. Images were analyzed in Image J, and surface profiles calculated with “Intensity Profile” (Schneider et al., 2012).

## Results

### Astrocyte Phagocytosis Under Flow Conditions

**FIGURE 1:**
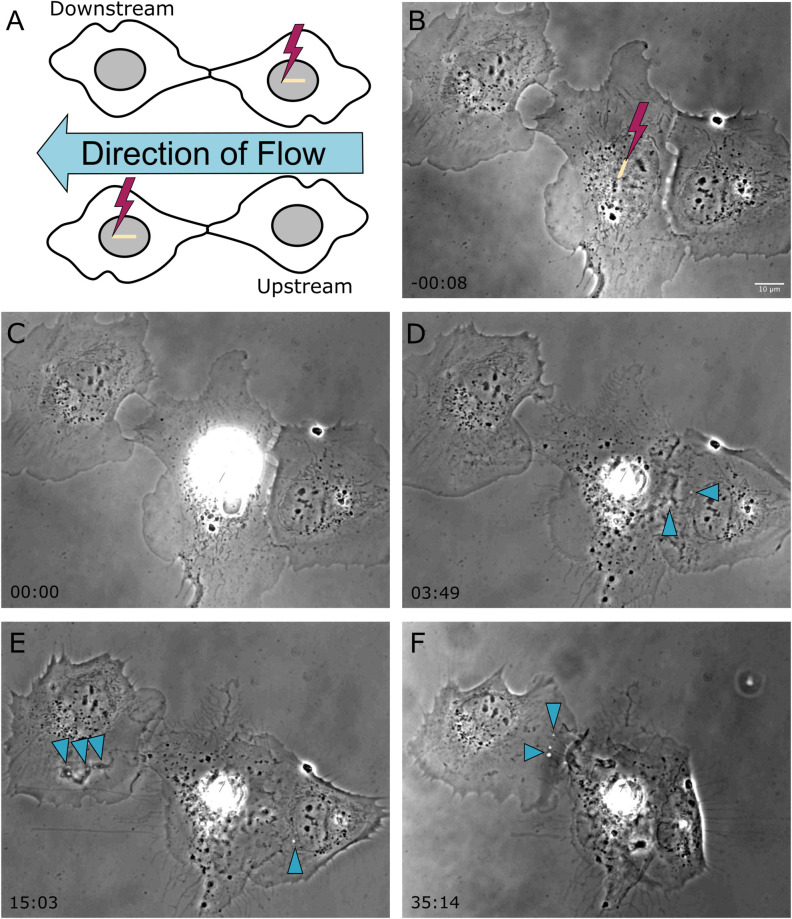
Shear stress was applied laterally from right to left on cells located horizontal to one another, as depicted in the schematic diagram in frame **(A)**. The cell located on the left is downstream of the irradiated cell, and the cell located on the right is upstream of the irradiated cell with respect the applied flow. **(B)** Prior to photolysis, astrocytes display no to minimal vesicles. The position targeted by the laser is depicted by the yellow ROI. **(C)** The laser is targeted to the nucleus of the central astrocyte as media flows laterally across the cells. **(D–F)** Phase contrast images show that the upstream cell initiated phagocytosis earlier than the downstream cell, as visible by cell ruffling and endocytic vesicle formation in image **(D)** acquired 3 min 49 s following photolysis for the downstream cell, and image **(E)** acquired 15 min 3 s for the upstream cell. Blue arrows highlight endocytic vesicles in phase contrast images of responding astrocytes.

**FIGURE 2:**
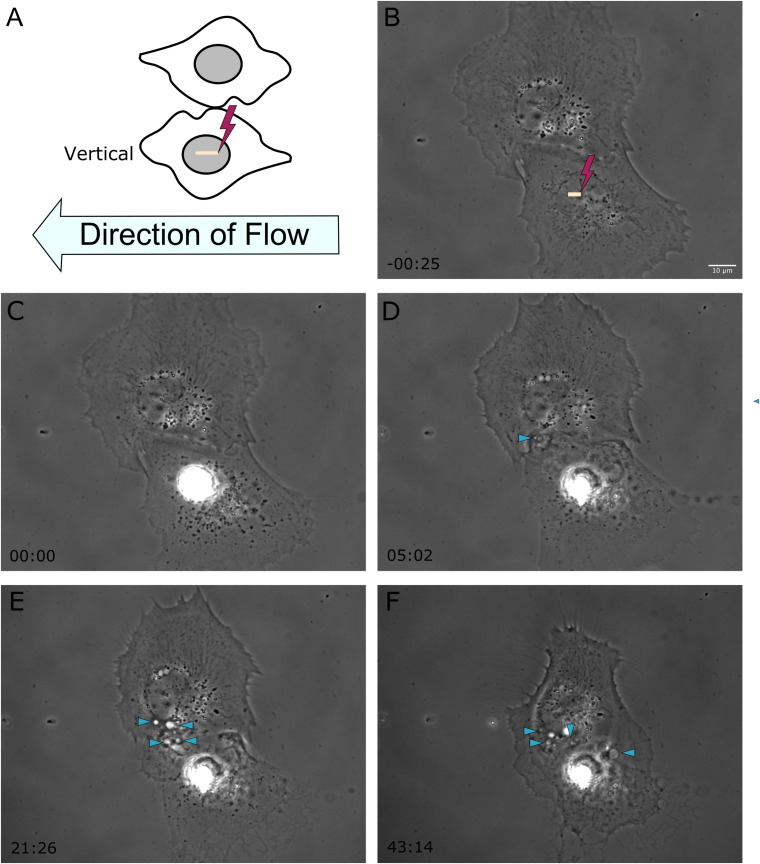
Shear stress was applied from right to left on astrocytes located vertical to one another, represented in frame **(A)**. The yellow ROI demarcates the position of photolysis in image **(B)**, acquired 25 s prior to laser exposure. The irradiated cell in this example is located below the observed responding astrocyte. **(C–F)** Phase contrast images show the initiation of phagocytosis and the formation of vesicles in the vertically positioned cell, with vesicles highlighted by blue arrow heads in images **(D–F)**, corresponding to 5 to 43 min post-photolysis.

In a previous study, we demonstrated the ability of astrocytes *in vitro* to phagocytose neighboring cellular debris released via targeted laser ablation (Wakida et al., 2018, 2020). In this study we elucidate the importance of mechanical forces on the phagocytic response of astrocytes by the application of laminar flow of media across the cell’s surface. With the use of laser ablation to lyse a targeted cell (photolysis), we observe astrocytes both prior to photolysis and during the phagocytic process of astrocytes (see Supplementary Movie 1). The imaging period prior to laser irradiation reveals a minimal number of vesicles visible within the cytoplasm of astrocytes (Figures 1, 2). A short-pulsed femtosecond laser was then used to photolyse the nucleus of a single astrocyte. The position of laser targeting that results in photolysis is indicated by the yellow region of interest (ROI) line. Responding cells are categorized based on the orientation to the cell. The first frame of Figure 1 denotes the position of responding cells, categorized as upstream and downstream with respect to the direction of lateral flow and the targeted cell.

In response to the lysed neighboring cell, membrane ruffling is immediately apparent as astrocytes respond to the lysed cell. The upstream cell in Figure 1 (cell to the right of the targeted cell) shows extensive changes to its cell morphology. Initially the upstream cell’s leading edge is oriented away from the targeted cell, and reverses its direction toward the targeted cell within 3:49 s following photolysis. At this time, small vesicles are visible near the cell periphery, as indicated by the blue arrows. The upstream cell continues to advance onto the lysed cell, covering the lysed cell almost completely by the end of the 36 min observation time. The downstream cell in Figure 1 (to the left of the targeted cell) responds in a similar fashion to the upstream cell, including membrane ruffling and reversing its leading edge to orient toward the lysed cell. The downstream cell’s phagocytic response time is much slower in comparison to the upstream cell, requiring reorientation its leading edge visible in image E of Figure 1 at 15:03 post-lysis, with formation endocytic vesicles (indicated by blue arrows) at its periphery at 15 (Figure 1E) and 35 min (Figure 1F).

**FIGURE 3:**
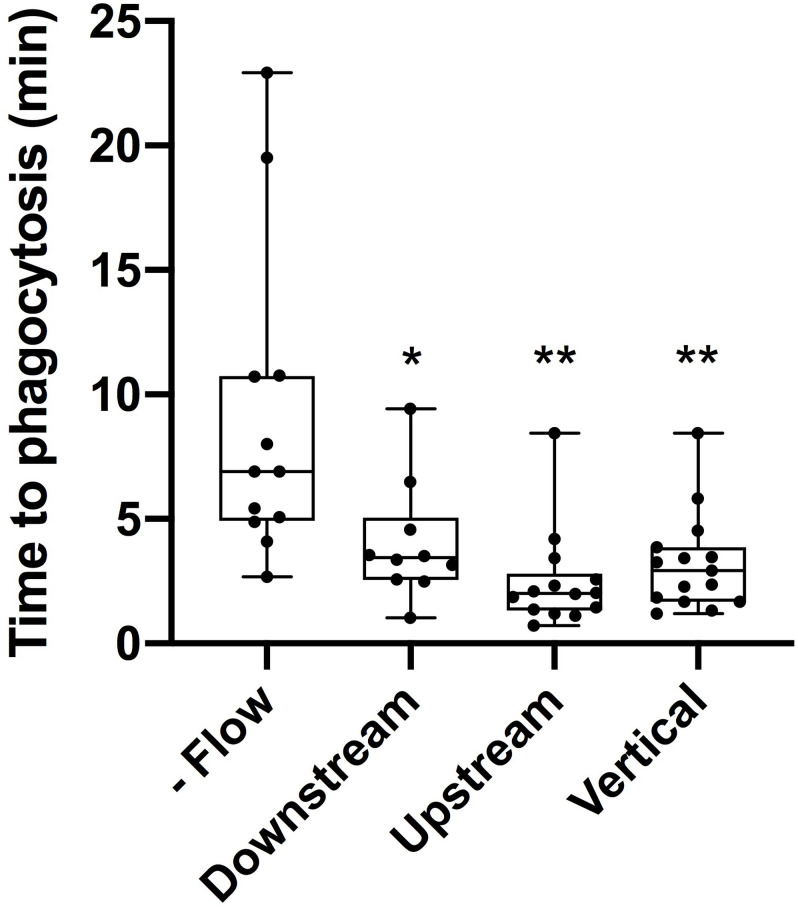
Cells under flow conditions initiated phagocytosis at a significantly faster rate than cells under static conditions. “No flow” cells under static conditions initiated phagocytosis on average 9 min post-photolysis. This was significantly longer (*p* = 0.028, denoted with 1 asterisk) than the average initiation time for downstream cells initiating phagocytosis on average 4 min post-lysis. Similarly, upstream cells with an average initiation time of 2.5 min and vertical cells with an average initiation time of 3.2 min both were significantly faster than no flow cells with *p* < 0.01 (denoted by 2 asterisks).

The average time to initiate phagocytosis was significantly shorter for cells under flow conditions in comparison to control astrocytes under static conditions (Figure 3). Control astrocytes responded via phagocytosis at a rate of 72% (*n* = 16), as determined by membrane ruffling and formation of vesicles at the cell periphery nearest the lysed cell. All cells observed under flow averaged a phagocytic rate of 3.1 min to initiation, significantly faster than phagocytosis initiated in static conditions, at an average of 9 min for the initiation of phagocytosis.

Responding astrocytes upstream of irradiated cell averaged 2.5 min to initiate a phagocytic response (*n* = 14), significantly faster in comparison to static cells (*p* < 0.01). Responding downstream astrocytes initiated phagocytosis on average 4 min following photolysis (*n* = 12), also significantly faster that control astrocytes, *p* < 0.01 (see Supplementary Tables 1, 2). Upstream cells responded on average 1.5 min faster to lysed cell debris than downstream cells, this difference was not significantly faster (*p* = 0.1).

To better understand the effect the shear flow of media had on the phagocytic response, cells vertically oriented to a lysed cell were observed by time lapse imaging (Figure 2). In this example, membrane ruffling and endocytic vesicles are clearly visible within the frame 2D 5 min and 20 s following photolysis, as indicated by the blue arrow. Dramatic cell morphology changes including the formation of endocytic vesicles at the cell’s periphery nearest the lysed cell continues over a 45 min observation period. The box plot in Figure 3 compare the time to initiating phagocytosis for all orientations of cells. Cells vertically oriented to the irradiated cell initiated phagocytosis on average 3.2 min following laser irradiation (*n* = 12). This was significantly faster in comparison to control astrocytes, *p* < 0.01. In comparison with upstream and downstream responding cells, there was no significant difference in response time for vertically oriented cells (upstream *p* = 0.33, downstream *p* = 0.36).

Astrocytes under flow conditions responded via phagocytosis at a significantly higher response rate of 95 vs. 72% of observed astrocytes in static conditions (*p* = 0.001 via 2-tailed chi-squared analysis). Astrocytes in static conditions (-Flow) displayed an inverse relationship between phagocytic response and distance between the laser ROI and responding cell’s plasma membrane (refer to Supplementary Figure 2). A phagocytic response of 100% was observed for responding astrocytes located 10–20 μm away from position of photolysis (ROI), decreasing to 91, 67, 40, and 17% for 20–30, 30–40, 40–50, and 50+ μm separation, respectively. Comparatively, astrocytes exposed to flow (+Flow) displayed a 100% phagocytic response for all astrocytes separated from 10 to 50 μm. Astrocytes in the presence of flow increased the phagocytic response regardless of the distance of separation from the photolysis ROI.

### Cell Forces

**FIGURE 4:**
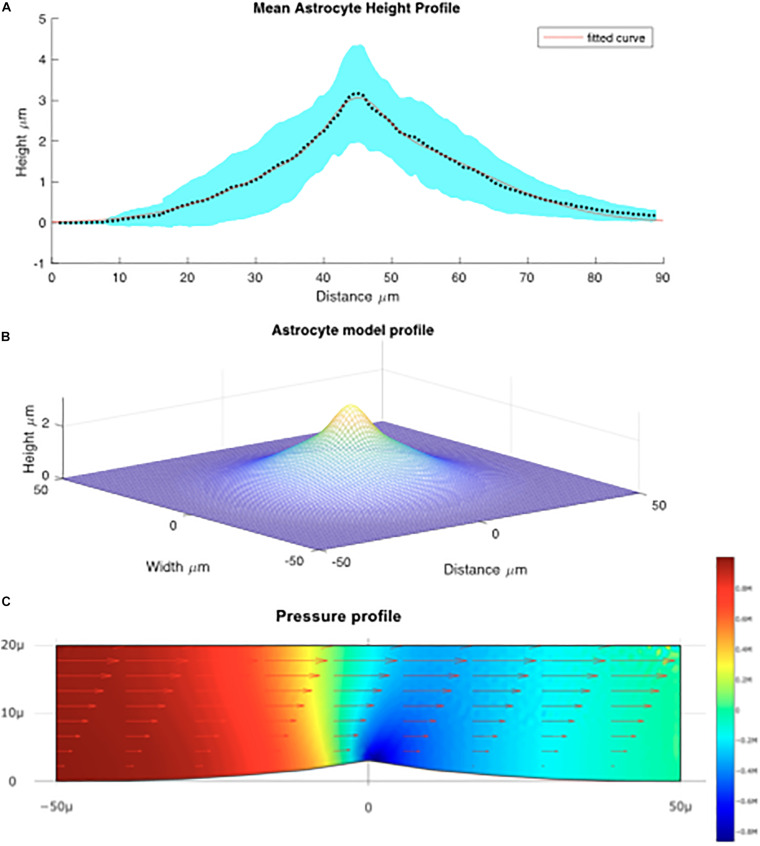
**(A)** Mean height profile of astrocytes derived from QPM data depicted by the dotted line. The red line denotes the fitted Gaussian function. The blue shaded area is the standard error derived from the QPM height profiles. **(B)** Model of the average astrocyte height profile used for modeling fluid flow over the cells. **(C)** Computed flow over the astrocyte cell. Arrows denote the fluid velocity, color denotes pressure.

For each of 16 astrocytes analyzed, the QPM was used to model a height profile along the maximal and minimal distances from the center of mass to the edge of the cell. The mean lengths of long and short lines were calculated separately; the mean for the short lines was approximately 32.25 μm and for the long lines 56.55 μm. Each array was scaled to the size of the mean using interpolation in MATLAB, and the mean of the thickness was calculated for short and long lines. Figure 4A shows the average size of the cells modeled.

Using this average astrocyte profile, a model of the “average cell” was created. It was found that the average size of the profile was best fit by a sum of Gaussian functions (see Figure 4B) in the form:

$$a\,e\,x\,p\,\left(-{\left(\frac{r}{b}\right)}^{2}\right)\,c\,e\,x\,p\,\left(-{\left(\frac{r}{d}\right)}^{2}\right)$$

where a = 1.05; b = 5.45; c = 2.04; d = 22.13;

In order to calculate the effect pressure and shear in the micro channel the fluid velocity was first calculated analytically using the formula. $U\,\left(y\right)\,\frac{6\,F_{R}}{b\,h^{3}}\,\left(h\,y-y^{2}\right)$

Here, b and h are the breadth and height of the chamber F_*R*_ is the flow rate and y is the distance from the base of the chamber. Note: Since the chamber was much longer than the entrance aperture it is assumed that a parabolic velocity distribution is present.

The shear force is given by:

$$\tau -\eta \,\frac{d\,U}{d\,y}$$

where η and is the viscosity water at 37°C. The system was then solved for the Navier-Stokes equations using finite element analysis. The computational fluid dynamics tool box was used to model flow around an idealized astrocyte cell (Figure 4C).

We can see that maximal force applied to the cell is located on the leading edge of the crest of the cell implying that the membrane surrounding the nucleus experiences large forces relative to the rest of the cell. Based on the vector field overlaid on Figure 4C which represents the fluid velocity, the applied shear force is greatest at the top most point of the cell, above the nucleus. This is because the change in fluid velocity is most pronounced at this point. We can also see from Figure 4C that the greatest lateral change in pressure occurs around the center of the cell which is indicative of a force being exerted. Though this analysis does not take into account structural elements within the cell or the deformability/rigidity of the cell structure it does imply that the applied force is greatest near the nucleus of the cell. We estimated the shear stress force on the cell will be about 0.025 Pa ± 10% depending on the position on the cell, or 0.25 dyn/cm^2^.

### Diffusion

In order to ascertain if flow alone can be responsible for the astrocyte behavior it is necessary to calculate the effects of diffusion alone on the system. Based on the Stokes-Einstein equation we can calculate that the diffusivity of calcium ions in water at 37°C, is 1.42 × 10^3^ μm^2^/s

where $D\,\frac{K_{B}\,T}{6\,\pi \,r\,\mu_{B}}$ where K_*B*_ = Boltzmann Constant, T = Temperature, μ_*B*_ Dynamic viscosity r = the van der Waals of radius 231 picometers. Given that the diffusion length is $L\,\sqrt{2\,D\,t}$ we can derive the inequality $v\,\frac{2\,D}{L}$. Thus in order for diffusion to overwhelm the fluid flow the flow must be greater than 328 μm/s. In practice the flows used in this experiment are significantly greater than this which flows in the channel reaching more than 4,000 μm/s. This analysis of course does not account for diffusion near the surface boundaries or for time dependent signaling. However, it is clear that for the most part the flow in the microfluidic should overwhelm diffusive effects.

## Discussion

In this study, we demonstrated the ability to combine laser nanosurgery with directed shear flow to better understand the phagocytic response of astrocytes. There is an increasing need to understand what environmental factors control the phagocytic process of astrocytes to clear cellular debris generated by an insult to the CNS. In a previous study, our lab demonstrated that astrocytes connected by a cytoplasmic process responded to a lysed cell at a significantly higher rate than isolated cells that do not share any membranous connection. Here, we demonstrate that the phagocytic response can be initiated at a significantly faster rate in the presence of shear fluid flow across the apical surface of astrocytes *in vitro*. Through calculations based on calcium ions, we confirmed the flow rate use was above the diffusion rate of calcium ions. We further measured the average height of astrocytes using QPM, to then model the shear force of fluid and pressure delivered to astrocytes during photolysis experiments. Pressure was found to be the highest at the nucleus, the subcellular location of photolysis.

Our calculations confirmed the flow rate aligned with our goal to create a flow of lysed material over the downstream cell, but not upstream cell. Modeling of shear forces demonstrate that forces are strongest at the site of photolysis, the nucleus. The initial goal of this study was to separate the mechanical pull of a lysed cell retracting from the diffusion of molecules from the lysed cell. To separate the mechanical and chemical signals believed to induce phagocytosis, media flowing unidirectionally across the cells’ apical surface was used to wash cell debris away from upstream cells. Cells downstream of the lysed cell were exposed to debris as media washed the photolysed debris over the responding cell. The response of the downstream cell would be affected by the chemical stimulation of debris washing over cells. The flow rate used in this experiment greatly exceeds the diffusion rate of cell debris. Calculations show based on the diffusion of calcium ions in water at 37°C, defined that flow must be greater than 328 μm/s. Thus, we assume cell debris is washed away from upstream cell, and only interacts with the downstream cell. The observed phagocytic response of the upstream cell would be dependent upon the mechanical stimulus of cell retraction following photolysis. The observation that upstream and downstream cells response time was not significantly different leads us to believe that diffusion of lysed cellular debris flowing over the cells is not the major contributing factor for membrane connected cells to react via phagocytosis. This finding provides a strong argument for a mechanical retraction of the lysed cell serving as an important signal in initiating the phagocytic response.

Surprisingly, all cells under flow conditions respond significantly faster than cells under static conditions. Astrocytes appear to be activated by shear flow, potentially allowing cells to respond with faster cytoskeletal remodeling. Results suggest that the direction of flow helps cell migrate, possibly by aiding actin pooling near the leading edge of the responding astrocyte. Cells which migrate in the opposite direction of media flow must fight against the shear force, thus resulting in a slightly delayed initiation of phagocytosis. This is also supported by upstream cells having the fastest average phagocytic initiation time, followed by vertically oriented cells, and slowest being the downstream cells. Analysis of astrocytic response based on the separation distance of the responding cell’s plasma membrane to the position of photolysis, showed that the addition of flow significantly increased the phagocytic response. Unlike the inverse relationship of distance with phagocytosis, all astrocytes under shear flow positioned 10–50 μm from photolysis responded via phagocytosis at a 100% rate (refer to Supplementary Figure 2). The combined observation of increased phagocytic response time and rate provides strong evidence that astrocytes can be activated by shear flow against or across the surface of the cell.

Our analysis of the forces created by continuous flow in a microfluidic channel shows media flowing across cells exerts significant force over the surface of the cell with most force concentrated near the nucleus. Though astrocytes *in vivo* would clearly be subjected to different range forces we have shown that in the simple case of constant flow a clear change in astrocyte response is evident. Other studies have dealt previously with oscillatory flow over cells, however, the change in astrocyte response with respect to the direction of the flow indicates that in this case flow direction is potentially significant. Through the current model is clearly useful in elucidating astrocyte behavior in the context of microfluidics more work needs to be done on the precise modeling of forces *in vivo.*

Our results suggest astrocyte response to neighboring cell damage or death is a mechanosensitive pathway. The rapid reorientation and phagocytosis observed in astrocytes aligns with previously reported observations of mechanically stimulated cytoskeletal rearrangement. Shear flow is known to induce motility in *Dicytostelium* (Décavé et al., 2003), where increased number of pseudopod extensions and actin reorganization have been observed. Cytoskeletal rearrangement in response to flow has been observed in mechanically stimulated neutrophils (Zhelev and Hochmuth, 1995) and widely observed in endothelial cells (Perrault et al., 2015). The time scale observed in astrocytes’ response aligns with the rapid response observed in endothelial cells to flow with increased traction forces and intercellular stress components followed by cell elongation and alignment along the direction of flow (Perrault et al., 2015; Steward et al., 2015). The time scale additionally aligns with reports by Vogel and Sheetz on cell motility responses due to mechanosensory transduction (Vogel and Sheetz, 2006). Relatedly, the shear stress force exerted on the cell was estimated to be 0.25 dyn/cm^2^. A study in neutrophils show response to lower shear forces than endothelial cell (below 1 Pa to stimulate neutrophils) similar to forces used to stimulate the immune-like response in astrocytes observed here (Janmey and Weitz, 2004). Cytoskeletal rearrangement is necessary for the migration and phagocytic response observed in shear flow activated astrocytes reported here.

Future studies will be 2-fold in expanding imaging and force measurement capabilities during shear flow experiments to reveal more information on the phagocytic process. Improved imaging techniques including confocal imaging would improve the resolution of subcellular events that may change in the presence of shear flow. Combining improved imaging with fluorescent labeling of potential contributors to the phagocytic process will be used to look at how signaling mechanisms is affected by the addition of shear flow. A previous study in epithelial cells demonstrate changes in calcium signaling in response to mechanical stimulation (Sanderson et al., 1990), as well as astrocytes (Charles et al., 1991). We have shown in previous studies significant calcium changes in astrocytes in response to photolysis (Wakida et al., 2020). Follow up studies will combine the application of shear flow to calcium imaging to better understand how intercellular signaling, including calcium, is affected by shear flow. Future studies would improve technology to precisely measure forces transmitted to responding cells by integrating laser doppler velocimetry or micro particle velocimetry. Improvement of both force measurements and imaging capabilities at higher resolutions will allow us to investigate the types and position of mechanosensitive receptors being activated during the observed.

Shear flow’s ability to enhance the phagocytic uptake of cellular debris demonstrates the potential to increase phagocytic uptake of cellular debris. It opens up the possibility of future studies where we can observe astrocyte behavior in response to stimulating mechanosensors like integrins or cadherins that are known to transduce mechanical stimuli (Sun et al., 2016; Yap et al., 2018). Finding ways to enhance the phagocytic response could have direct implications on the study of brain injury. Decreasing secondary injury to nervous tissue via enhanced phagocytic uptake of cellular debris could directly increase the quantity of surviving neurons.

## Data Availability Statement

The datasets for this study can be found in the figshare repository 10.6084/m9.figshare.12827795.

## Ethics Statement

The animal study was reviewed and approved by the Brain Bits animal protocol #32-08-013 was approved by the Southern Illinois University School of Medicine Laboratory Animal Care and Use Committee.

## Author Contributions

NW and DP designed experiments for this study and wrote the manuscript. NW, GC, PP, and DP executed experiments, data analysis, statistical testing, calculations, and modeling. MB and DP provided critical feedback and direction of project. All authors contributed to the article and approved the submitted version.

## Conflict of Interest

The authors declare that the research was conducted in the absence of any commercial or financial relationships that could be construed as a potential conflict of interest.

## Supplementary Material

Supplementary Figure 1 Schematic diagram of combined pump system, laser optical path, and microscopy system.

Supplementary Figure 2 Phagocytic response of astrocytes based on separation from laser ROI. Laser ROI to closest plasma membrane of respond astrocyte.

Supplementary Table 1 The average time to phagocytosis for the control (Brain bits – connected) and the average time to phagocytosis for cells located downstream, upstream, and vertical from the irradiated cells were recorded.

Supplementary Table 2 Comparison of the average time to phagocytosis between different conditions. There was a significant difference (*p* > 0.05) in the average time to phagocytosis between the control and the application of shear stress. There was also significant differences between the average time to phagocytosis between thecontrol and the upstream, downstream, and vertical conditions.

Supplementary Movie 1 Astrocyte phagocytosis under lateral shear flow.
