# Supplementary figures and images for: Fluid Shear Stress Enhances the Phagocytic Response of Astrocytes
Source: Front Bioeng Biotechnol. 2020 Nov 11;8:596577. doi: 10.3389/fbioe.2020.596577 (PMC7686466; doi:10.3389/fbioe.2020.596577)

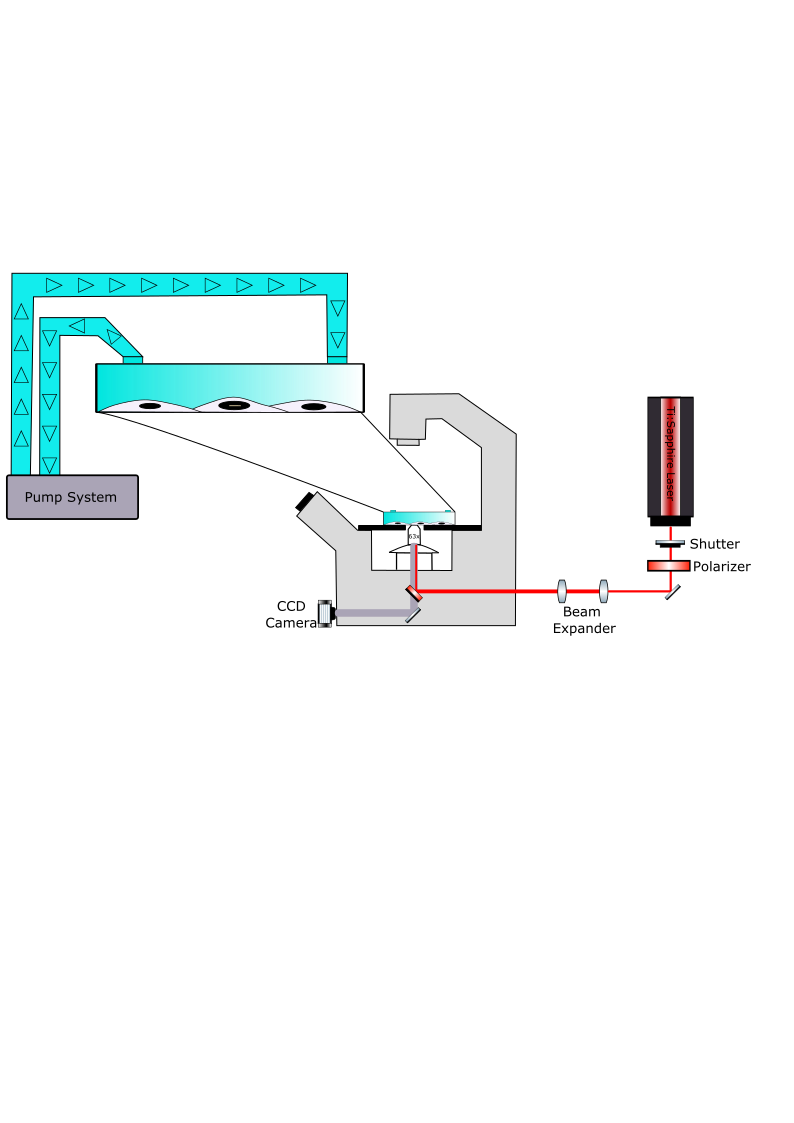

Supplement: Supplementary Figure 1 — Schematic diagram of combined pump system, laser optical path, and microscopy system. [file Image_1.JPEG]

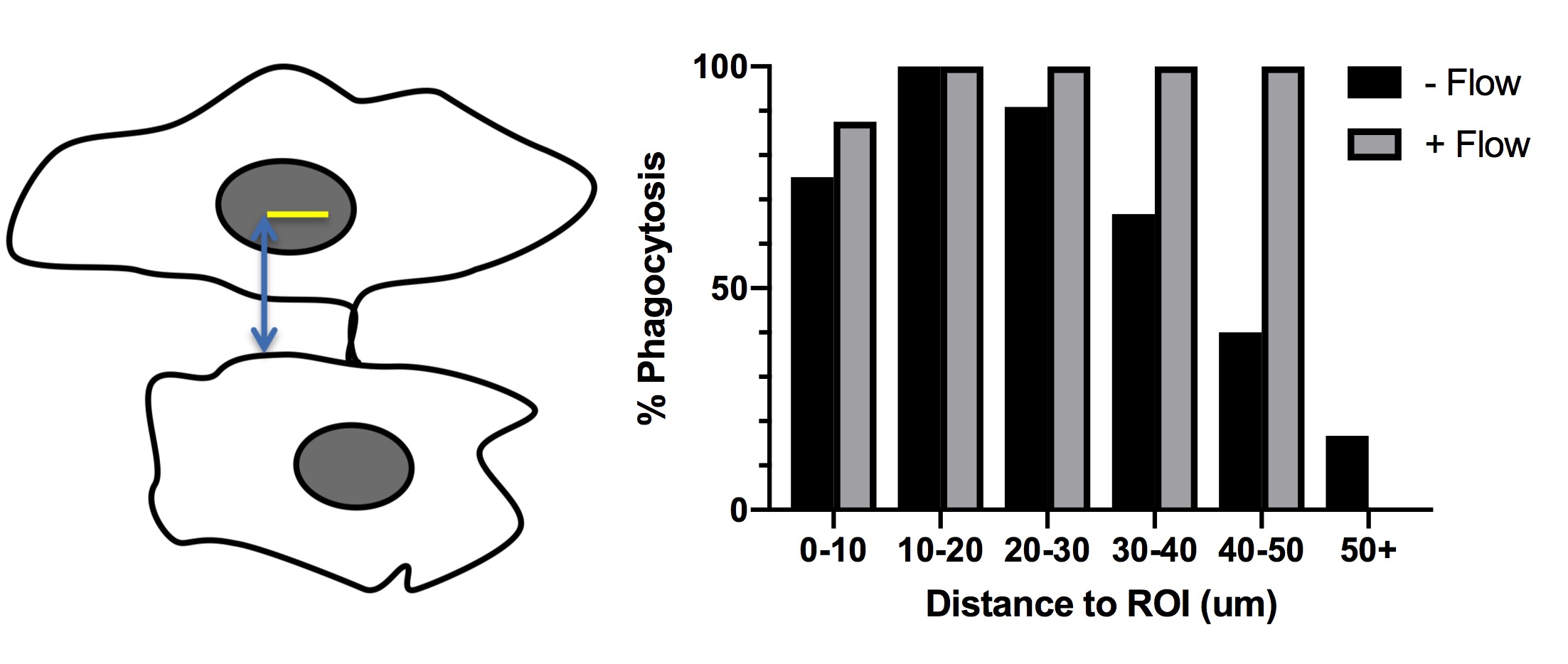

Supplement: Supplementary Figure 2 — Phagocytic response of astrocytes based on separation from laser ROI. Laser ROI to closest plasma membrane of respond astrocyte. [file Image_2.JPEG]
