# Supplementary material for: Fluid Shear Stress Enhances the Phagocytic Response of Astrocytes
Source: Front Bioeng Biotechnol. 2020 Nov 11;8:596577. doi: 10.3389/fbioe.2020.596577 (PMC7686466; doi:10.3389/fbioe.2020.596577)
Supplement: Supplementary Table 1 — The average time to phagocytosis for the control (Brain bits – connected) and the average time to phagocytosis for cells located downstream, upstream, and vertical from the irradiated cells were recorded. [file Presentation_1.PPTX]

## Slide 1
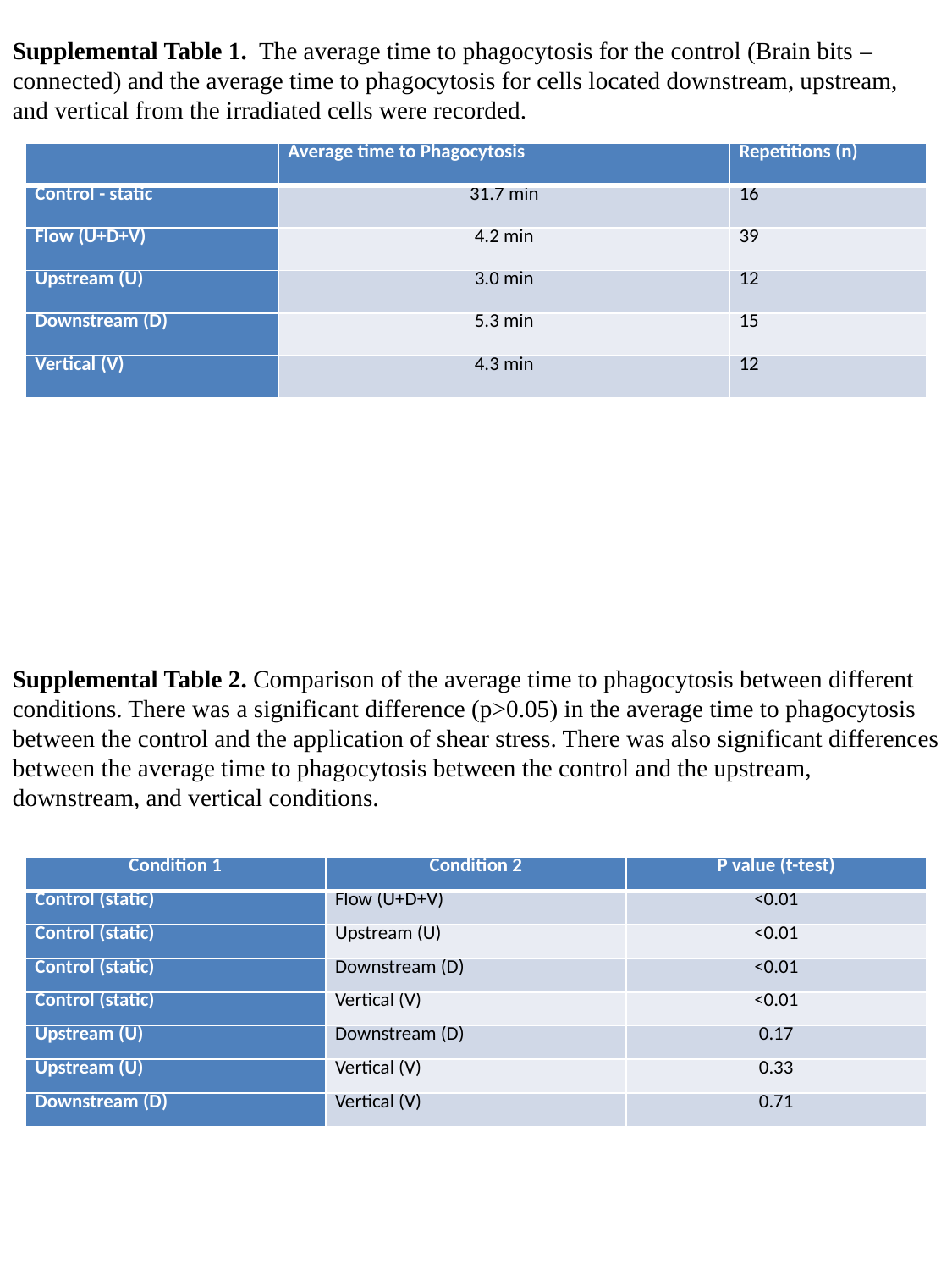

Supplemental Table 1. The average time to phagocytosis for the control (Brain bits – connected) and the average time to phagocytosis for cells located downstream, upstream, and vertical from the irradiated cells were recorded.
| | Average time to Phagocytosis | Repetitions (n) |
| --- | --- | --- |
| Control - static | 31.7 min | 16 |
| Flow (U+D+V) | 4.2 min | 39 |
| Upstream (U) | 3.0 min | 12 |
| Downstream (D) | 5.3 min | 15 |
| Vertical (V) | 4.3 min | 12 |
Supplemental Table 2. Comparison of the average time to phagocytosis between different conditions. There was a significant difference (p>0.05) in the average time to phagocytosis between the control and the application of shear stress. There was also significant differences between the average time to phagocytosis between the control and the upstream, downstream, and vertical conditions.
| Condition 1 | Condition 2 | P value (t-test) |
| --- | --- | --- |
| Control (static) | Flow (U+D+V) | <0.01 |
| Control (static) | Upstream (U) | <0.01 |
| Control (static) | Downstream (D) | <0.01 |
| Control (static) | Vertical (V) | <0.01 |
| Upstream (U) | Downstream (D) | 0.17 |
| Upstream (U) | Vertical (V) | 0.33 |
| Downstream (D) | Vertical (V) | 0.71 |
